# Genotype × Herbivore Effect on Leaf Litter Decomposition in *Betula Pendula* Saplings: Ecological and Evolutionary Consequences and the Role of Secondary Metabolites

**DOI:** 10.1371/journal.pone.0116806

**Published:** 2015-01-26

**Authors:** Tarja Silfver, Ulla Paaso, Mira Rasehorn, Matti Rousi, Juha Mikola

**Affiliations:** 1 Department of Environmental Sciences, University of Helsinki, Niemenkatu 73, FI-15140 Lahti, Finland; 2 The Finnish Forest Research Institute, Vantaa Research Unit, FI-01301 Vantaa, Finland; Umeå Plant Science Centre, Umeå University, SWEDEN

## Abstract

Plant genetic variation and herbivores can both influence ecosystem functioning by affecting the quantity and quality of leaf litter. Few studies have, however, investigated the effects of herbivore load on litter decomposition at plant genotype level. We reduced insect herbivory using an insecticide on one half of field-grown *Betula Pendula* saplings of 17 genotypes, representing random intrapopulation genetic variation, and allowed insects to naturally colonize the other half. We hypothesized that due to induced herbivore defence, saplings under natural herbivory produce litter of higher concentrations of secondary metabolites (terpenes and soluble phenolics) and have slower litter decomposition rate than saplings under reduced herbivory. We found that leaf damage was 89 and 53% lower in the insecticide treated saplings in the summer and autumn surveys, respectively, which led to 73% higher litter production. Litter decomposition rate was also affected by herbivore load, but the effect varied from positive to negative among genotypes and added up to an insignificant net effect at the population level. In contrast to our hypothesis, concentrations of terpenes and soluble phenolics were higher under reduced than natural herbivory. Those genotypes, whose leaves were most injured by herbivores, produced litter of lowest mass loss, but unlike we expected, the concentrations of terpenes and soluble phenolics were not linked to either leaf damage or litter decomposition. Our results show that (1) the genetic and herbivore effects on *B. pendula* litter decomposition are not mediated through variation in terpene or soluble phenolic concentrations and suggest that (2) the presumably higher insect herbivore pressure in the future warmer climate will not, at the ecological time scale, affect the mean decomposition rate in genetically diverse *B. pendula* populations. However, (3) due to the significant genetic variation in the response of decomposition to herbivory, evolutionary changes in mean decomposition rate are possible.

## Introduction

Aboveground herbivores typically consume 5–20% of plant biomass in terrestrial ecosystems [1, 2]. They can also affect ecosystem processes through various direct and indirect mechanisms [3–6] and depending on how plants respond to herbivory, herbivores can either accelerate or decelerate nutrient cycling [5–7]. For instance, if plant species that are preferred by herbivores can tolerate herbivory through compensatory growth of nitrogen (N) rich tissues, rapid decomposition of litter may result, which together with herbivore waste products accelerates nutrient cycling [5–6, 8]. In contrast, deceleration of nutrient cycling can occur when the preferred, N-rich plant species cannot tolerate the herbivory and the well-defended species that produce litter of low quality increase in dominance [5–6, 9–10]. Many plant species, such as the fast growing deciduous woody plants, also produce a vast array of chemical compounds in their foliage in response to herbivory [11–12] and these compounds can remain through senescence and decelerate subsequent litter decomposition [13–14]. Because leaf litter fall forms an important stock of nutrients in terrestrial ecosystems [15–16], such herbivore-induced changes in the quantity and quality of litter can have significant effects on nutrient cycling rates [6].

It is commonly assumed that herbivores and decomposers are repelled or attracted by similar plant traits [17–19] and among plant species the palatability of leaves to generalist herbivores is positively correlated with litter decomposition rate [19–21]. It is reasonable to assume that a similar association is found among plant genotypes, which like plant species, can considerably vary in herbivore resistance, leaf quality and litter decomposability [22–26]. However, two studies only have so far examined the genetic link between herbivore palatability and litter decomposition. Schweitzer et al. [14] found no genetic link between the abundance of leaf-galling aphids and litter decomposition among five *Populus* spp. genotypes and our own study with 19 *Betula pendula* genotypes suggests that the association is opposite to that found among plant species, i.e. leaf litter mass loss is highest for those genotypes that are least damaged, or least preferred, by herbivores during the growing season [27].

Recent research has demonstrated that genetic variation in dominant species, like trees, can have distinct effects on the structure and functioning of their ecosystems [28–31]. Understanding the link between herbivory and litter decomposition rate at tree genotype level will further reveal how tree adaptation to herbivory and the following changes in the genetic structure of tree populations can affect nutrient dynamics in ecosystems. However, while convincing examples of life and afterlife effects of genes have been presented in many studies [28–31], it has been argued that the role of genetic variation may be largely overestimated [32–33]. This is because many studies have concentrated on study systems with particular ecological characteristics, such as hybrid zones. Most studies are also confounded by a common methodological flaw, i.e. the genotypes are collected from diverse and distant environments to maximize the genetic variation, while the experiments are performed in a single common garden, where the environmental variation is minimal [32–33]. This argument calls for studies that deliberately contrast the genetic variation of local populations with the spatial variation of their local environments.

Here, we address the need for studies that are implemented at local spatial scales and examine the ability of insect herbivores and intrapopulation genetic variation to affect leaf litter decomposition in local forest stands. Our study species, *B. pendula*, is one of the most common European tree species [34] and is particularly abundant in the northern and eastern parts of Europe [35]. It is a typical pioneer tree species, which can rapidly colonize open forest patches [34, 36] and dominate the early successional stages of boreal forests. To avoid the flaw of maximizing the genetic variation while minimizing the environmental variation, we selected random *B. pendula* genotypes from a naturally regenerated 0.9-ha forest stand and planted their vegetatively produced progeny to a similar 0.6-ha clear-cut forest site [37]. In our earlier study [27], we hypothesized that our finding of litter mass loss being lowest for those genotypes, which were least resistant to insect herbivores, was due to induced defence: i.e. those genotypes, whose leaves were most injured during the growing season (possibly due to weakest intrinsic resistance), invested most in the induced defence, which was then manifested as low leaf litter mass loss.

In the present study, we tested the hypothesis that herbivores decelerate litter decomposition by inducing secondary metabolite production [13–14] at both the population and the genotype level. Of the secondary metabolites, we focused on terpenes and soluble phenolics because their concentrations have been shown to respond to insect herbivory in *B. pendula* [38–39]. We reduced the herbivore load on one half of the saplings of each genotype using an insecticide, while the insects naturally colonized the other half, and predicted that (1) those saplings that are subjected to natural, higher herbivore load produce litter of lower quality, in terms of higher concentrations of terpenes and soluble phenolics, and have smaller litter mass loss than seedlings subjected to reduced herbivore load. Such a response would tell of a general, population level response of leaf litter decomposition to herbivory. However, we predicted that (2) this pattern should also emerge among *B. pendula* genotypes, i.e. those genotypes, whose leaves are most damaged (i.e. have low intrinsic resistance), produce litter of lowest quality due to induced defence and have smallest litter mass loss. Such genotypic correlations would suggest that the mean decomposition rate in the population can be modified by natural selection and might, for instance, increase if selection in the future, due to higher herbivore load in warmer climate [40], would favour intrinsic herbivore resistance.

## Materials and Methods

### Study site, plant material and the insecticide treatment

The genotypes used in this study were originally selected from a naturally regenerated mixed *B. pendula—B. pubescens* Ehrh. forest stand in Punkaharju, south-east Finland (61°48’ N, 29°18’ E) to investigate the intrapopulation genetic variation of plant traits in *B. pendula* [22]. The saplings used in the present study were micropropagated from these trees, or their cloned progeny, at the Haapastensyrjä Unit of the Finnish Forest Research Institute (FFRI) in early 2008. The new plantlets were grown in a nursery and overwintered in a cold room before planted on a FFRI owned field site in Loppi, South Finland (60°36’N, 24°24’E; 140 m a.s.l.) in the spring 2009. The soil in the field site is post-glacial sorted fine sand, topped by a humus layer of a few centimetres thick, and has a pH of 5.0 and C and N concentrations of 6% and 0.3%, respectively, in the top 5 cm layer [37]. The history, weather and ground layer vegetation of the site are described in Mikola et al. [37]. The field study did not involve endangered or protected species and did not require any specific permission.

The field site is divided into six replicate blocks, each with 132 planting plots, and the saplings of different genotypes are randomly allocated to these plots [37]. For this study, two saplings of each of the 17 genotypes were randomly selected from each block in 2011: one was used as a control and weekly sprayed with water (being thus naturally colonized by insects), while the other was weekly sprayed with 0.1% solution of the synthetic pyrethrin insecticide Decis EC25 (Bayer CropScience, Germany) [41]. Portable garden sprayers (one for pyrethrin, the other for water) were used for the sprayings and a shower cubicle was placed around the sapling while spraying to control the wind drift of the insecticide. In our earlier laboratory assessment, the 0.1% Decis EC25 solution had no side effects on *B. pendula* growth, or the chemistry of green leaves and leaf litter [41].

### Measuring leaf damage, litter chemistry and litter mass loss

To estimate the effect of the insecticide on herbivore load and the genotypic variation of herbivore damage (an estimate of plant resistance), leaf damage was monitored at the top and at the second highest side branch of each sapling in the middle (late July) and end (middle of September) of the growing season. Leaf damage was scored using a modified Schreiner-type method [42], in which a damage index ranging from 0 to 100 is produced by multiplying two scores: A, which gives the average area damaged per leaf (0 = 0%, 1 = 1–4%, 5 = 5–20% and 25 = 21–100% of leaf area eaten) and B, which gives the percentage of damaged leaves (0 = 0%, 1 = 1–25%, 2 = 26–50%, 3 = 51–75% and 4 = 76–100% of all leaves damaged). To estimate sapling growth, the height of the saplings was measured in early spring and late autumn and the growth was then calculated as height increment.

Leaf litter was collected by enclosing each sapling in a mesh bag before autumn leaf abscission. The bags were collected from the field after all leaves had fallen from all saplings. After measuring the total fresh mass of the litter, eight to ten leaves were randomly picked from each bag, weighed and placed in a 10×10 cm litter mesh bag (mesh size 0.5 mm). These bags were returned to the field site in late November and laid on the ground surface, all in one randomly chosen spot. The litter that was placed in the litterbags was not dried for dry mass measurements in order to preserve the microbes, such as endophytes [43], which grow on the falling litter. Instead, a subsample of eight leaves was picked from each litter collector bag. These subsamples were dried and their water concentrations were used to estimate the initial litter dry mass in each litter bag, as well as the total litter production for each sapling. The litterbags were collected from the field at the end of August 2012, dried (at 60°C for three days), weighed and the percentage loss of litter mass was calculated for each sapling.

To investigate the insecticide effect on litter quality (i.e. the concentrations of terpenes, soluble phenolics, C and N), six leaves, corresponding to 0.3 g dry mass, were collected from each litter collector bag in 2011. The leaves of each genotype were pooled for the insecticide and control treatments (n = 1 for genotype in both treatments) and ground in liquid N. Litter chemistry has a mechanistic role in this study, and for this purpose (i.e. to reveal genotypic associations with herbivore damage and litter decomposition) genotype means are adequate. Litter N and C concentrations were analysed using a LECO CNS-2000 Analyzer (LECO Corporation, USA), while terpenes and soluble phenolics were analysed using a Waters Acquity UPLC system (Waters, Milford MA, USA), attached to interfaced Waters Synapt G2-S HDMS mass spectrometer (details for the extraction using MeOH and analysis using Acquity UPLC BEH C18 column are given in Silfver et al. [41]). The metabolites were identified using retention times, UV spectra and HPLC-MS, and their concentrations are relative, i.e. given as peak area g^−1^ dry litter material. A full list of the analysed compounds, their alignment into different compound groups and mean concentrations in each treatment are in S1 Table.

### Statistics

The broad-sense heritabilities (*H^2^*) of leaf damage index, leaf litter production and litter mass loss were calculated on individual plant basis according to the equation 1, where $\sigma_{G}^{2}$, $\sigma_{B}^{2}$, $\sigma_{GxI}^{2}$and $\sigma_{E}^{2}$are variance components for genotypes, blocks, genotype × insecticide interaction and (within-block) environment, respectively. The variance components were calculated using the SPSS GLM Variance components (REML) procedure. The insecticide treatment was included in the variance component models as a fixed factor. Coefficients of genotypic variation (*CV_G_*) were further calculated according to the equation 2, where $\bar{X}$is the phenotypic mean.

$$H^{2}=\sigma_{G}^{2}/\left(\sigma_{G}^{2}+\sigma_{B}^{2}+\sigma_{GxI}^{2}+\sigma_{E}^{2}\right)$$ (1)

$$CV_{G}=\sqrt{\sigma_{G}^{2}}/\bar{X}$$ (2)

The effects of *B. pendula* genotype and the insecticide treatment on leaf damage index, litter production and litter mass loss were statistically tested using the General Linear Models (GLM)-procedure of the SPSS statistical package (version 20.0.0.1; IBM SPSS Statistics). In the models, the genotype was treated as a random factor and the insecticide treatment as a fixed factor. The replicate block was included in the models as a random factor. Moreover, since the leading shoots of some saplings were damaged in the spring (i.e. they were dark brown/black or dry and the buds were not swollen) and this can significantly affect sapling growth and performance [37], the saplings were classified as damaged or not damaged and this grouping was included in the models as an independent two-level fixed factor. Leaf litter production was logarithm transformed to equalize the error variances for the GLM analyses. The effects of the insecticide treatment on litter concentrations of N, C and the secondary metabolites were tested using a pairwise t-test and pooled genotype values. The genotypic correlations among plant and litter attributes were estimated using the Pearson correlation coefficients of genotype means. The data that were used in the statistical analyses are in supporting information file S1 Data.

## Results

**Figure 1:**
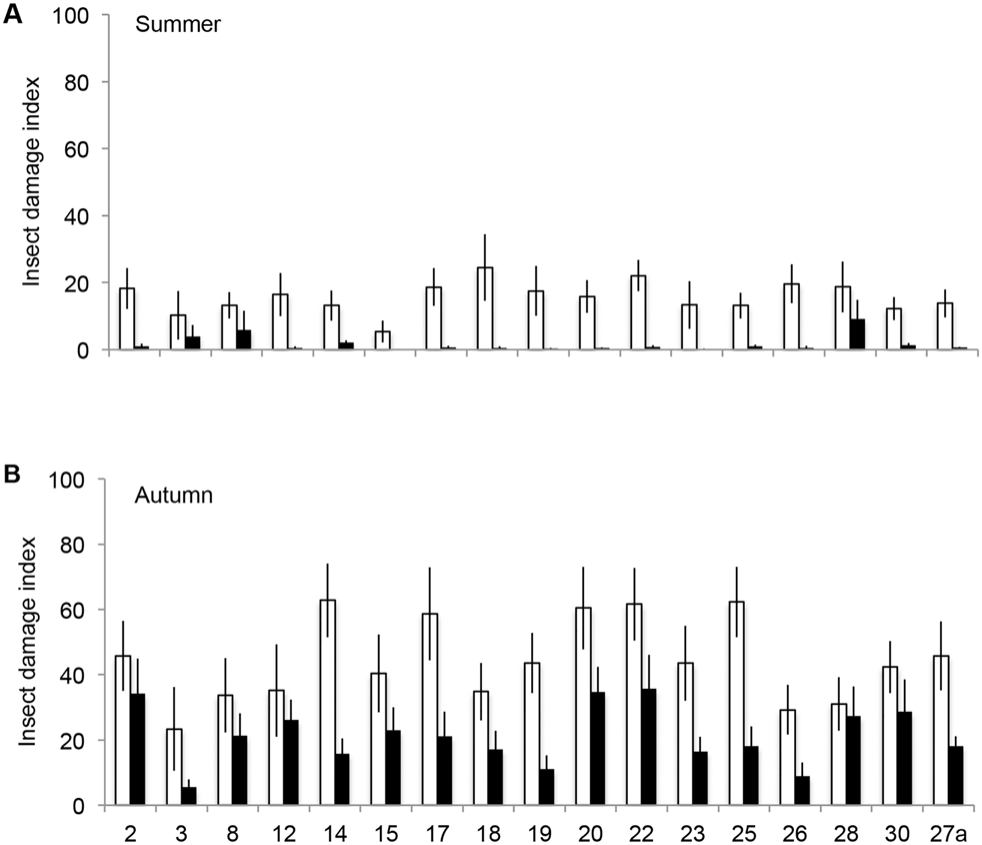
Summer and autumn leaf damage indices of the insecticide treated (black bars) and control (white bars) saplings of 17 *B. pendula* genotypes (mean±SE, n = 5–6 for each bar).

The insecticide treatment decreased the leaf damage of the *B. pendula* saplings on average by 89 and 53% in the summer and autumn surveys, respectively (Fig. 1, Table 1). In the autumn survey, leaf damage also differed significantly among the genotypes (Fig. 1, Table 1) and 7.7% of the total phenotypic variation in herbivore damage was explained by the genotypic variation (Table 2). The field block did not explain the phenotypic variation in leaf damage in either survey (Table 2).

**Table 1:** The ANOVA table of the effects of genotype and insecticide on *B. pendula* insect herbivore damage index, litter production and litter mass loss.

	***df***	***F***	***p***
*Summer DI*			
Genotype	16, 16	0.66	0.827
Insecticide	1, 16	119	**< 0.001**
G × I	16, 159	0.68	0.808
Block	5, 159	0.19	0.966
*Autumn DI*			
Genotype	16, 16	1.82	**0.033**
Insecticide	1, 16	59.1	**< 0.001**
G × I	16, 158	0.87	0.606
Block	5, 158	0.17	0.972
*Litter production*			
Genotype	16, 16	1.30	0.206
Insecticide	1, 16	38.4	**< 0.001**
G × I	16, 154	0.52	0.931
Block	5, 154	3.98	**0.002**
LSD 2009	1, 154	2.05	0.155
*Litter mass loss*			
Genotype	16, 16	1.10	0.357
Insecticide	1, 16	<0.01	0.956
G × I	16, 154	1.73	**0.047**
Block	5, 154	5.92	**< 0.001**
LSD 2009	1, 154	5.11	**0.025**

**Table 2:** Variance components (*σ^2^*), broad-sense heritability (*H^2^*), phenotypic mean ($\bar{X}$) and the coefficient of genotypic variance (CV_G_) of *B. pendula* insect herbivore damage index (DI), litter production and litter mass loss (G = genotype, B = block, I = insecticide, E = error, or residual).

	**$\sigma_{G}^{2}$**	**$\sigma_{B}^{2}$**	**$\sigma_{GxI}^{2}$**	**$\sigma_{E}^{2}$**	***H^2^***	**$\bar{X}$**	**CV_G_**
Summer DI	0	0	0	108.7	0	8.72	0
Autumn DI	37.62	0	0	483.1	0.072	32.9	0.186
Litter production	0.002	0.007	0	0.068	0.026	0.58	0.077
Litter mass loss	0	9.01	2.63	55.71	0	10.8	0

**Figure 2:**
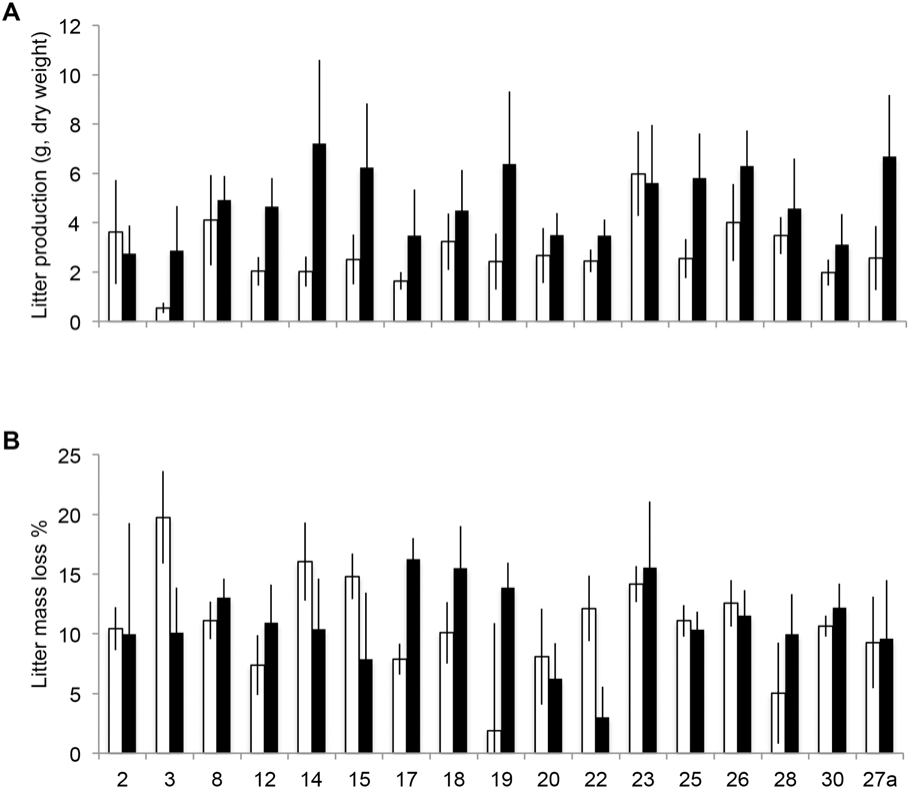
Leaf litter production and litter mass loss of the insecticide treated (black bars) and control (white bars) saplings of 17 *B. pendula* genotypes (mean±SE, n = 4–6 for each bar).

Leaf litter production was increased by the insecticide treatment on average by 73% (Fig. 2, Table 1) and the effect was equal among the genotypes as the phenotypic variation was not explained by the genotype × insecticide interaction (Table 1, 2). The variation among field blocks in leaf litter production was statistically significant (Table 1) and explained 9.1% of the total phenotypic variation (Table 2). The genotype effect, in turn, was insignificant and explained only 2.6% of the total variation (Table 2).

In contrast to the effect on leaf litter production, the genotype × insecticide effect on litter mass loss was statistically significant (Fig. 2, Table 1) and explained 3.9% of the total phenotypic variation (Table 2). As a consequence, the broad-sense heritability for litter mass loss was zero (Table 2) and the insecticide main effect was not significant (the mean mass loss for control and insecticide-treated saplings was 10.6 and 11.0%, respectively) (Fig. 2, Table 1). The field block, where the litter had been produced, had a very significant influence on litter mass loss (Table 1) and explained 13.4% of the total phenotypic variation (Table 2).

Litter C concentration was minimally, but statistically significantly, increased by the insecticide treatment, while the N concentration and C/N-ratio were unaffected (Table 3). The concentrations of terpene and soluble phenolic groups were generally higher in the insecticide-treated than control saplings, but the difference was statistically significant for quercetin and kaempferol glycosides only (Table 3).

**Table 3:** Means (±SE) of the attributes of leaf litter chemistry (the concentrations of secondary compounds are relative, i.e. given as peak area g^−1^ dry litter material) in the insecticide treated and control saplings and the statistical significance of the difference between the means (tested using a pairwise *t*-test).

	**Treated**	**Control**	***P***
C%	53.0±0.1	52.6±0.1	**0.014**
N%	1.22±0.03	1.30±0.06	0.124
C/N	43.9±1.2	41.7±2.0	0.213
(+)-catechin	0.94±0.42	0.65±0.17	0.495
DHPPG	2.80±1.32	8.68±3.40	0.124
Cinnamic acid derivatives	18.5±6.3	12.7±3.8	0.459
Myricetin glycosides	87.1±34.3	38.7±14.7	0.173
Quercetin glycosides	804±143	400±63	**0.012**
Kaempferol glycosides	55.9±5.1	38.4±5.1	**0.005**
Flavonoid aglycones	2630±209	2423±197	0.415
Salicylates	4.39±1.31	4.32±0.95	0.962
Sum of phenolic compounds *	3603±297	2926±215	0.078
Triterpenes	576±140	406±32	0.239

* Inlcudes (+)-catechin, DHPPG, cinnamic acid derivatives, flavonol glycosides (myricetins, quercetins and kaempferols), flavonoid aglycones and salicylates.

**Figure 3:**
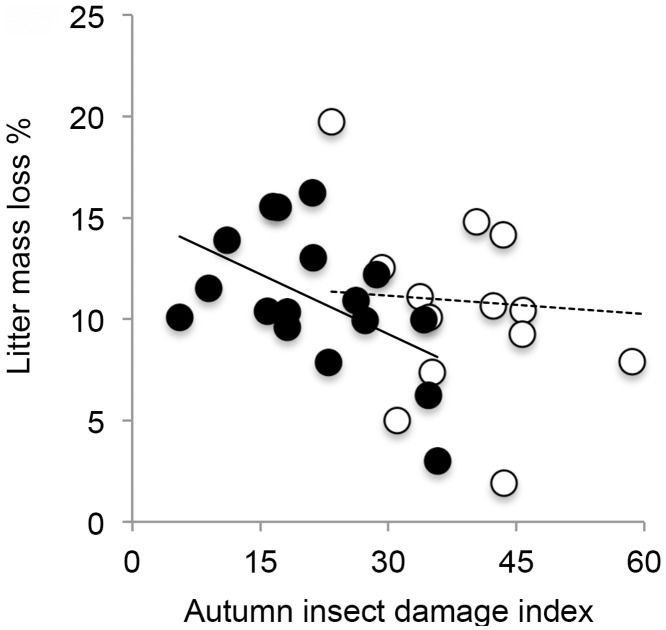
Correlation of genotype means of autumn insect damage index and litter mass loss for control (white dots) and treated (black dots) seedlings.

The genotype means of litter mass loss and leaf damage were negatively correlated in the insecticide-treated, but not in the control saplings (Fig. 3, Table 4). The litter chemistry attributes (N % and the concentrations of terpenes and soluble phenolics) and the sapling growth estimates (height growth and litter production) were not genetically linked to either leaf damage or litter mass loss, except for the DHPPG concentration, which was lowest in those genotypes that were most damaged (Table 4).

**Table 4:** Pearson correlation coefficients of genotype means of plant traits, autumn damage index (treated and control saplings combined because the genotype × insecticide effect was insignificant) and litter mass loss (separately for treated and control saplings because the genotype × insecticide effect was significant).

	**Autumn damage index**		**Litter mass loss**			
			***Control saplings***		***Treated saplings***	
	**r**	***P***	**r**	***P***	***r***	***P***
Litter C%	−0.11	0.692	0.05	0.862	−0.15	0.578
Litter N%	−0.13	0.641	0.39	0.132	0.17	0.536
Litter C/N-ratio	0.18	0.511	−0.30	0.257	−0.18	0.501
(+)-catechin	−0.06	0.813	0.11	0.675	0.05	0.870
DHPPG	**−0.58**	**0.020**	0.19	0.473	0.01	0.960
Cinnamic acid derivatives	−0.08	0.760	0.26	0.328	−0.12	0.663
Myricetin glycosides	0.04	0.890	−0.19	0.479	0.04	0.880
Quercetin glycosides	0.14	0.605	0.24	0.381	−0.05	0.864
Kaempferol glycosides	−0.18	0.516	0.37	0.162	0.06	0.821
Flavonoid aglycones	−0.15	0.581	0.10	0.701	−0.13	0.634
Triterpenes	0.02	0.957	0.24	0.377	−0.07	0.808
Salicylates	−0.30	0.263	−0.26	0.330	0.02	0.931
Sum of phenolic compounds	−0.07	0.787	0.04	0.876	0.08	0.773
Height growth	0.14	0.604	−0.34	0.183	0.22	0.399
Litter production	−0.17	0.507	−0.09	0.741	0.13	0.605
Autumn damage index			−0.09	0.728	**−0.52**	**0.034**

## Discussion

We predicted that *B. pendula* saplings would produce leaf litter with higher concentrations of terpenes and soluble phenolics, and consequently lower mass loss, when growing under natural than reduced herbivory. Our insecticide treatment decreased leaf damage by 53–89%, which shows that the herbivore load was significantly reduced, but our prediction was falsified: the litter produced under reduced herbivore pressure had generally higher concentrations of terpenes and soluble phenolics and the insecticide effect on litter mass loss varied from negative to positive among the genotypes. In contrast to studies, in which the herbivore-induced secondary metabolites remained in the litter and decelerated litter decomposition [13–14], our results suggest that higher herbivore pressure may decrease the production of terpenes and soluble phenolics in *B. pendula* saplings and further, that there is no clear link between these compounds and the decomposition rate of *B. pendula* litter. The same pattern also emerged at the genotype level: we found significant genotypic variation in leaf damage, but the intensity of damage was not related to terpene or soluble phenolic concentrations (except for DHPPG, which had the lowest concentrations in the genotypes with the highest damage) and there was no genotypic correlation between the concentrations of these metabolites and litter mass loss. The most damaged genotypes produced litter that had lowest mass loss as we also found earlier [27], although this association was statistically significant for the insecticide-treated saplings only.

That concentrations of secondary metabolites, and particularly those of quercetin and kaempferol glycosides, were lower when the herbivore load was higher indicates that our saplings did not primarily produce these compounds for herbivore defence. We have earlier shown that our insecticide treatment does not have side effects on green leaf or leaf litter chemistry in *B. pendula* [41], which guarantees that the higher concentrations in the insecticide-treated saplings were due to lower herbivore load, not due to direct chemical effects. The production of many phenolics can be induced by insect herbivory in *Betula* species [38–39, 44], but they can also perform as antioxidants against environmental threats, such as elevated UVB-radiation [45–46], and many of those phenolic compounds (e.g. quercetin 3-galactoside/glucoside and kaempferol 3-rhamnoside), which we found in higher concentrations in our insecticide-treated saplings (S1 Table) have earlier been shown to be induced by UVB-radiation [45–46]. In our field site, the saplings compete heavily with ground layer vegetation, which is manifested by their slow growth rate [37], and herbivory further reduces the growth [41]. In such harsh conditions, unlike in more favourable laboratory or nursery conditions, where the induction of secondary compound production has earlier been documented [38, 44–46], the production may simply follow the availability of C resources, not the level of herbivory. This argument follows the logic in the carbon-nutrient balance hypothesis [47], which predicts that the production of carbon-based secondary metabolites increases when carbohydrates accumulate in plant tissues. In our study, the availability of C resources was apparently greater in the insecticide-treated saplings as they produced litter of higher C concentration and their growth was 44% higher than the growth of saplings subjected to natural herbivory [41]. Most likely these elevated C resources permitted the higher production of secondary compounds. Generalizing our findings, however, requires caution. We studied young saplings, and in bigger trees, which are better able to compete with ground layer vegetation and whose growth may not be as adversely affected by herbivore feeding, the allocation of C resources to secondary metabolites may not follow the same pattern.

Using a meta-analysis, Carmona et al. [48] recently showed that secondary compounds are not among the plant traits that best predict the variation of herbivore resistance among plant species. This is against the widely accepted belief that secondary metabolites have a central role in plant herbivore resistance [49–52]. Our results point to the same direction at plant genotype level as we found practically no association between herbivore resistance (revealed by leaf damage) and terpene and soluble phenolic concentrations among the *B. pendula* genotypes. Moreover, against the hypothesis that secondary metabolites can explain the intra- and interspecific variation in litter decomposition [18–19, 24, 26], we found no genetic link between the concentrations of our metabolites and litter mass loss. It should be noted though that we did not determine the concentrations of insoluble phenolics, i.e. condensed tannins. These compounds have earlier been shown to retard soil microbial activity and decelerate decomposition [18, 53] and their concentrations do not decrease during leaf senescence as much as those of soluble phenolics [54]. On the other hand, earlier studies have shown that concentrations of condensed tannins are not generally induced by defoliation in *B. pendula* [38] and our own recent study (Silfver et al., unpublished manuscript) shows that the genotypic variation in *B. pendula* litter mass loss cannot be explained by the variation in the concentrations of condensed tannins. This suggests that neither the insoluble nor soluble phenolics are the mechanism that could link herbivore pressure and the genetic variation in leaf damage and leaf litter decomposition in *B. pendula*.

We found significant genotypic variation for insect damage and litter mass loss, and the broad-sense heritability and coefficient of genotypic variance for the damage index were close to those earlier calculated for height growth (*H^2^* = 0.055 and CV_G_ = 0.21) using the same plant material [37]. For litter mass loss, the *H^2^* and CV_G_ were zero, but this was due to significant genotypic variation in the response of litter decomposition to varying herbivore load, which explained 3.9% of the total phenotypic variation. Our heritability estimates for litter decomposition rate are much lower than those earlier observed for *Populus tremuloides* (30% of the total variation in decomposition explained by the genotype) in a study, where the genotypes were selected from geographically distinct areas [26]. This supports the idea that the importance of genetic variation in ecosystem functioning may be overestimated in the current literature because of the mismatch of the spatial scales of the genetic and environmental variation [33]. On the other hand, our results show that significant genetic effects on ecosystem functioning can also emerge in a heterogeneous environment, and particularly, when there is no mismatch in the scale of genetic and environment variation. This supports the view that genetic variation in dominant species can have distinct effects on the functioning of their ecosystems [28–31].

In our field site, the replicate blocks capture the spatial variation of soil organic matter content and ground layer vegetation. In our study year, the block explained 6.9% of total phenotypic variation in sapling growth and the difference in mean growth between the best and worst block was 2.1-fold [37]. These effects on growth logically explain the significant block effect on leaf litter production in our study, but more interesting is that the block also had a very clear effect on litter mass loss, explaining 13.4% of the total phenotypic variation. Since the litterbags were placed near to each other in one of the replicate blocks, this effect was entirely created during leaf growth and senescence and could be related to the significant block scale variation in green leaf N concentrations in our study site [37]. In the light of these effects, the insignificant block effect that we found on leaf damage was unexpected. Besides leaf N concentrations, leaf water concentrations vary significantly among the blocks in our site [37] and insect herbivores should prefer leaves of high N and water concentrations [55–56]. The evidence that herbivores follow such local variation comes from a recent *B. pendula* study, where the structure of insect communities varied significantly among the field blocks [57].

Herbivory effects on litter dynamics vary a lot among tree species: for example, herbivory increased the annual litter inputs and accelerated the litter decomposition of coniferous *Pinus edulis* [58], while in deciduous *Populus* species the litter decomposition rate was decelerated [14]. In our study, herbivory was very intensive, 12-fold in comparison to an earlier *B. pendula* study which used the same leaf damage index [23], and leaf litter production was on average 73% higher under reduced than natural herbivore load. The effect of herbivore pressure on litter decomposition, however, varied among the genotypes, and because the effects ranged from positive to negative, the effect faded to insignificance at the population level. This suggests that increasing insect herbivory rates in the future warmer climate [40] are likely to reduce leaf litter production, but may not influence the average litter decomposition rate in *B. pendula* populations, providing that the populations remain genetically diverse.

To conclude, our results underline the value of investigations at tree genotype level when searching for the link between herbivores, trees and forest ecosystem functioning. At the population level and at the ecological time scale, our results suggest no effect of insect herbivore load on leaf litter decomposition. However, looking at this link at the genotype level reveals significant genetic variation in the response of litter decomposition to herbivore load. This will permit changes in the mean litter decomposition rate in populations at the evolutionary time scale if higher herbivore load in the future climate [40] will favour genotypes, which have on average higher or lower litter decomposition rate. What complicates the prediction of such changes in *B. pendula* populations is that we could not find the mechanism that we sought for to explain the observed intrapopulation genotypic link between herbivory resistance and leaf litter decomposition in *B. pendula*. All our evidence suggests that secondary compounds, and terpenes and soluble phenolics particularly, are not the missing mechanism.

## Supporting Information

S1 Data Herbivore damage index, growth, litter production and litter mass loss in 17 different *B. pendula* genotypes planted on six field blocks. Insect herbivory was reduced using an insecticide on one half of the saplings while local insects naturally colonized the other half. The Excel data file also includes litter chemistry data for control and insecticide-treated genotypes.
(XLSX)

S1 Table Identification parameters and mean (±SE) concentrations (peak area g^−1^ dry litter material) of secondary metabolite compounds in the insecticide treated and control *Betula pendula* saplings and the statistical significance of the difference between the means (n = 16, i.e. the samples were pooled within genotypes). (DOCX)
