# Supplementary material for: Genotype × Herbivore Effect on Leaf Litter Decomposition in Betula Pendula Saplings: Ecological and Evolutionary Consequences and the Role of Secondary Metabolites
Source: PLoS One. 2015 Jan 26;10(1):e0116806. doi: 10.1371/journal.pone.0116806 (PMC4306545; doi:10.1371/journal.pone.0116806)
Supplement: S1 Table — (DOCX) [file pone.0116806.s002.docx]

Table S1. Identification parameters and mean (±SE) concentrations (peak area g^-1^ dry litter material) of secondary metabolite compounds in the insecticide treated and control *Betula pendula* saplings and the statistical significance of the difference between the means (n=16, i.e. the samples were pooled within genotypes).

| Compound | | **Ion(s)** | **Treated** | **Control** | **P** |
| --- | --- | --- | --- | --- | --- |
|  | DHPPG | 329, 167 | 2.80±1.32 | 8.68±3.40 | 0.124 ^a^ |
|  | (+)-catechin | 291, 147 | 0.94±0.42 | 0.65±0.17 | 0.495 |
| *Cinnamic acids* | |  |  |  |  |
|  | Coumaroyl-quinic acid 1 | 339, 147 | 5.60±1.86 | 3.26±1.37 | 0.345 |
|  | Chlorogenic acid | 355, 163 | 3.46±1.79 | 2.96±0.83 | 0.814 |
|  | Coumaroyl-quinic acid 2 | 339, 147 | 4.96±2.36 | 3.36±1.24 | 0.557 |
|  | Coumaroyl-quinic acid 3 | 339, 147 | 4.46±1.22 | 3.10±1.01 | 0.355 |
| *Flavonol glycosides* | |  |  |  |  |
|  | Myricetin 3-galactoside | 481, 319 | 30.9±12.47 | 14.2±4.98 | 0.078 |
|  | Myricetin 3-glucuronide | 495, 319 | 1.38±0.69 | 0.66±0.39 | 0.274 |
|  | Myricetin 3-glucoside | 481, 319 | 14.0±5.04 | 4.45±1.53 | **0.033** |
|  | Myricetin 3-arabinopyranoside | 451 | 8.00±4.79 | 1.74±0.75 | - |
|  | Myricetin 3-arabinofuranoside | 451, 319 | 20.0±13.66 | 9.45±7.65 | - |
|  | Myricetin 3-rhamnoside | 465, 319 | 12.8±8.84 | 6.94±3.42 | - |
|  | Quercetin 3-galactoside ^b^ | 465, 303 | 649±118 | 323±52.0 | **0.011** |
|  | Quercetin 3-glucoside ^b^ | 465, 303 | 32.9±7.83 | 13.5±3.53 | **0.029** |
|  | Quercetin 3-arabinofuranoside | 435, 303 | 12.3±3.65 | 5.36±1.64 | 0.110 |
|  | Quercetin 3-arabinopyranoside ^b^ | 435, 303 | 30.4±17.9 | 10.0±5.57 | - |
|  | Quercetin 3-rhamnoside ^b^ | 449, 303 | 74.7±18.5 | 44.9±11.6 | 0.172 |
|  | Quercetin 3-glucuronide ^a^ | 479, 303 | 2.68±0.81 | 2.01±0.51 | 0.434 |
|  | Quercetin 3-rutinoside | 611, 303, 147 | 2.07±0.41 | 1.46±0.41 | 0.316 |
|  | Kaempferol 3-arabinofuranoside | 287 | 13.4±1.72 | 7.95±1.30 | **0.032** |
|  | Kaempferol 3-glucuronide | 463, 287 | 0.49±0.10 | 0.56±0.12 | 0.695 |
|  | Kaempferol 3-rhamnoside ^a^ | 287 | 11.2±2.39 | 5.60±1.11 | **0.025** |
|  | Kaempferol 3-glu/galactoside | 771, 449, 287 | 11.9±1.44 | 7.14±0.99 | **0.018** |
|  | Kaempferide-glucoside | 463, 301 | 18.9±3.24 | 17.2±2.55 | 0.503 |
| *Flavonoid aglycones* | |  |  |  |  |
|  | Flav 345_1 | 345 | 14.3±3.30 | 13.4±2.43 | 0.755 |
|  | Cirsimarin | 477, 315 | 159±14.5 | 127±16.8 | 0.051 |
|  | Apigenin | 271, 153 | 296±31.2 | 262±35.5 | 0.300 |
|  | Flav 301_1 | 654, 301 | 475±43.0 | 496±45.9 | 0.660 |
|  | Flav 331_1 | 331 | 74.7±8.37 | 76.0±6.70 | 0.861 |
|  | Flav 347 | 347 | 29.0±3.99 | 21.2±2.97 | 0.126 |
|  | Flav 345_2 | 345 | 8.30±1.38 | 6.38±0.88 | 0.201 |
|  | Flav 301_3 | 301 | 14.8±2.44 | 13.1±2.56 | 0.523 |
|  | Flav 331_2 | 331 | 48.0±8.08 | 43.5±9.53 | 0.518 |
|  | Flav 361_1 | 361 | 200±17.6 | 191±18.3 | 0.661 |
|  | Flav 331_3 | 331 | 17.8±4.45 | 9.42±1.81 | 0.064 |
|  | Flav 345_3 | 345 | 381±52.2 | 419±44.2 | 0.425 |
|  | Flav 361_2 | 361 | 28.6±4.30 | 20.4±2.69 | 0.147 |
|  | Flav 345_4 | 345 | 15.1±2.78 | 12.0±2.54 | 0.290 |
|  | Flav 375 | 375 | 116±12.3 | 99.8±12.8 | 0.207 |
|  | Flav 285, acacetin | 285 | 345±44.4 | 265±27.3 | 0.089 |
|  | Acacetin | 285 | 5.24±3.71 | 5.06±3.52 | - |
|  | Flav 331_4 | 331 | 55.9±8.75 | 34.0±5.17 | **0.050** |
|  | Flav 315_2 | 315 | 74.7±12.51 | 76.0±11.5 | 0.934 |
|  | Flav 345_5 | 345 | 266±22.9 | 232±33.5 | 0.229 |
|  | Flav 315_3 | 315 | 4.48±1.09 | 4.93±1.52 | 0.808 |
| *Triterpenes* | |  |  |  |  |
|  | Trit 527 | 527 | 225±44.5 | 166±15.1 | 0.198 |
|  | Trit 603_1 | 603 | 2.88±1.65 | 0.93±0.45 | - |
|  | Trit 341 | 341 | 213±52.5 | 140±15.2 | 0.191 |
|  | Trit 603_2 | 603 | 23.5±8.44 | 14.5±4.34 | 0.378 |
|  | Trit 279 | 279 | 11.6±2.03 | 8.02±1.33 | **0.019** |
|  | Trit 515/555 | 555 | 49.8±6.86 | 53.3±8.35 | 0.514 |
|  | Trit 557 | 557 | 0.19±0.06 | 0.07±0.04 | - |
|  | Trit 571 | 571 | 38.3±30.82 | 16.1±7.40 | 0.481 |
|  | Trit 545 | 545 | 12.5±6.24 | 7.07±5.48 | - |
| *Salicylates* | |  |  |  |  |
|  | Salicylate der | 139 | 0.57±0.24 | 0.38±0.15 | - |
|  | Salicylate | 139 | 1.27±0.84 | 1.24±0.50 | - |
|  | Saligenin | 125 | 0.01±0.01 | 0.30±0.12 | - |
|  | Salicortin 1 | 425 | 1.49±0.42 | 1.09±0.34 | 0.191 |
|  | Salicortin 2 | 425 | 1.34±0.39 | 1.36±0.45 | 0.979 |

- = not tested, because the compound was detected in less than half of the litter samples

^a^ the compound was induced by enhanced UVB radiation in a study by Tegelberg et al [45]

^b^ the compound was induced by enhanced UVB radiation in a study by Keskisaari et al. [46]
